# Collapsing Focal Segmental Glomerulosclerosis in Viral Infections

**DOI:** 10.3389/fimmu.2021.800074

**Published:** 2022-01-13

**Authors:** Anne K. Muehlig, Sydney Gies, Tobias B. Huber, Fabian Braun

**Affiliations:** ^1^ III. Department of Medicine, University Medical Center Hamburg-Eppendorf, Hamburg, Germany; ^2^ University Children’s Hospital, University Medical Center Hamburg-Eppendorf, Hamburg, Germany; ^3^ University Children’s Research@Kinder-UKE, University Medical Center Hamburg-Eppendorf, Hamburg, Germany

**Keywords:** podocyte, APOL 1, HIVAN, COVAN, Immune response

## Abstract

Collapsing glomerulopathy represents a special variant of the proteinuric kidney disease focal segmental glomerulosclerosis (FSGS). Histologically, the collapsing form of FSGS (cFSGS) is characterized by segmental or global condensation and obliteration of glomerular capillaries, the appearance of hyperplastic and hypertrophic podocytes and severe tubulointerstitial damage. Clinically, cFSGS patients present with acute kidney injury, nephrotic-range proteinuria and are at a high risk of rapid progression to irreversible kidney failure. cFSGS can be attributed to numerous etiologies, namely, viral infections like HIV, cytomegalovirus, Epstein–Barr-Virus, and parvovirus B19 and also drugs and severe ischemia. Risk variants of the APOL1 gene, predominantly found in people of African descent, increase the risk of developing cFSGS. Patients infected with the new Corona-Virus SARS-CoV-2 display an increased rate of acute kidney injury (AKI) in severe cases of COVID-19. Besides hemodynamic instability, cytokine mediated injury and direct viral entry and infection of renal epithelial cells contributing to AKI, there are emerging reports of cFSGS associated with SARS-CoV-2 infection in patients of mainly African ethnicity. The pathogenesis of cFSGS is proposed to be linked with direct viral infection of podocytes, as described for HIV-associated glomerulopathy. Nevertheless, there is growing evidence that the systemic inflammatory cascade, activated in acute viral infections like COVID-19, is a major contributor to the impairment of basic cellular functions in podocytes. This mini review will summarize the current knowledge on cFSGS associated with viral infections with a special focus on the influence of systemic immune responses and potential mechanisms propagating the development of cFSGS.

## Introduction

Focal segmental glomerulosclerosis (FSGS) is a major glomerular cause of end stage renal disease. The definition of FSGS is derived from its histopathological picture—the focal appearance of segmental scarring in some glomeruli. Before sclerosis ensues, podocytes show foot process effacement, leading to the manifestation of proteinuria. Since FSGS represents a pattern of response to injury, it was recently proposed to group FSGS into primary (immune-mediated) FSGS, adaptive FSGS, FSGS caused by pregnancy-related VEGF-inhibition, genetic, drug- and virus-associated FSGS (1, 2). The Columbia classification of FSGS differentiates 5 morphologic variants, namely, FSGS not otherwise specified (NOS), perihilar, cellular, tip, and collapsing variant (3).

The collapsing form of FSGS (cFSGS) represents a special form of secondary FSGS. Histopathologically, cFSGS is characterized by segmental or global condensation and obliteration of the capillary tuft associated with wrinkling and collapsing of the glomerular basement membrane. The podocytes display a distinct hyperplastic and hypertrophic phenotype, often containing cytoplasmic protein resorption droplets and pronounced foot process effacement. Severe tubulointerstitial disease with inflammation, edema, interstitial fibrosis and tubular atrophy as well as tubular regenerative changes constitutes an important component of cFSGS (4). cFSGS is associated with different etiologies. One of the best characterized causes is an infection with the human immune deficiency virus (HIV) and the development of HIV-associated nephropathy (HIVAN) (5). Furthermore, cFSGS can also be attributed to other infections, drugs, severe ischemia, autoimmune disease, genetic causes, and idiopathic (6–9). Additionally, an infection with the new Corona-Virus during the pandemic of SARS-CoV2 has been associated with the potential to develop cFSGS.

## HIV-Associated cFSGS

HIV infection accompanied by acute kidney injury, proteinuria, and a rapid progression to irreversible kidney failure characterizes the course of HIVAN. Tubuloreticular aggregates in endothelial cells and microcystic tubular dilatation in some cases may contribute to differentiate HIVAN from other etiologies of cFSGS in light microscopy (7).

Investigating the interaction of the virus or viral gene products with podocyte signaling pathways that induce massive morphologic alterations in cFSGS, might contribute to our understanding of podocyte biology and find a targeted therapy in (collapsing) FSGS independent of its etiology. It has been shown, even though podocytes do not express CD4-receptors or other known HIV-receptors, that podocytes are directly infected by HIV (10). The virus is known to damage the actin cytoskeleton in any cell type (11). The podocyte cytoskeleton is essential for the maintenance of the glomerular filtration barrier.

Furthermore, it has been shown, that HIV-1 also induces vascular endothelial growth factor (VEGF), leading to proliferation and de-differentiation of podocytes in cFSGS (12). Podocyte VEGF overexpression in a mouse model was able to cause glomerular disease with podocyte foot process effacement (13), while it was also shown that VEGF is crucial for podocyte survival *via* phospatidyl inositol 3 kinase/Akt signaling (14).

HIV associated cFSGS predominantly affects patients of African descent carrying a risk variant of the Apolipoprotein L gene 1 (APOL1), termed G1 and G2. The only known physiological function of APOL1 is its anti-trypanosomal activity (15). Several subspecies of trypanosoma have developed resistency against the “normal” G0 variants of APOL1. Therefore, the presence of one of the APOL1 variants G1 or G2 appears to protect against infection of several subspecies of *Trypanosoma brucei* (16–18). APOL1 is an abundantly secreted protein that circulates and associates with apolipoprotein A-I as a component of high-density lipoprotein (HDL) (19). It is also expressed in the intracellular compartment of various tissues—in the kidney specifically in glomeruli, proximal tubular endothelia and arteriolar endothelium. Within healthy glomeruli, APOL1 is localized exclusively in the podocytes (20). In both FSGS and HIVAN however, APOL1 expressing α-smooth muscle actin-positive cells were detected in the media of medium arteries and arterioles.

The APOL1 risk alleles G1 and G2 increase the risk of chronic kidney disease and are associated with an elevated risk of developing hypertension-associated end stage renal disease, FSGS, Lupus-nephritis, and HIVAN (16, 21–23).

Numerous studies suggested multiple pathways leading to impaired podocyte function and injury in HIVAN. The lack of the APOL1 gene in most model organisms and the absence of tissue specificity constitute barriers in identifying the underlying mechanisms. Conversely, the lack of the APOL1 gene in most mammals and a case report of a healthy APOL1-null patient supports the hypothesis, that APOL1 is not essential for kidney development and homeostasis (24).

The overexpression of APOL1 risk variants in podocytes was associated with increased necrosis and lysosomal permeability with leakage of lysosomal enzymes like cathepsin L into the cytosolic and nuclear compartment. Cathepsin L-induced degradation of the cytoskeletal protein F-actin might contribute to podocyte injury (25). Reduced numbers of autolysosomes led to impaired autophagic flux in APOL1 risk variant expressing HEK293 cells and podocytes (26). APOL1 risk variant dependent on upregulation of miR193a was found to result in the dedifferentiation of podocytes by blocking autophagy (27). Furthermore, APOL1 risk variants seem to downregulate expression levels of nephrin and podocin, key players in the slit diaphragm, and mediators of crucial signaling pathways (28).

These studies indicated the important role of APOL1 in the development of FSGS, as podocyte loss due to cell death occurred in all models of APOL1 risk variant overexpression. Pyroptosis, but not apoptosis was found to be increased in APOL1 risk allele transfected cells and might be a result of elevated levels of cleaved caspase 1 (26).

Scientific efforts could show that expression of human APOL1 risk variants in kidneys, spleens, and macrophages of bacterial artificial chromosome (BAC) transgenic mice promotes cholesterol accumulation (29). BAC/APOL1 transgenic mice express either the G0 allele or the risk alleles G1 and G2 under the endogenous APOL1 promotor. In line with these results, a recent study demonstrated in an APOL1/BAC mouse model that APOL1 risk variant expression drives lipid accumulation in renal cortices but not proteinuria. APOL1 risk variant expression in transgenic mice of a FSGS model (APOL1; Podocin-rtTA; NFATc1^nuc^) were more susceptible to doxycyclin induction as their wildtype littermates (WT; Podocin-rtTA; NFATc1^nuc^) and developed podocyte loss and mesangial matrix expansion. Interestingly, at baseline the human APOL1 transgenic mice did not develop proteinuria. The investigation of urinary podocytes from FSGS patients carrying either the G0/G0 or G1/G2 allele suggested that the APOL1 risk variants cause mitochondrial dysfunction linked with lipid accumulation and compensatory OXPHOS complexes elevation in podocytes (30).

Since most carriers of two risk alleles do not develop kidney disease spontaneously and the APOL1 variant expression itself was not sufficient to induce podocyte dysfunction, a second hit is postulated essential for the development of kidney disease. Infection with HIV is believed to be the strongest known risk factor for APOL1-associated kidney disease (18) as the innate immune response to HIV upregulates APOL1 gene expression (31).

Several innate immune pathways like interleukin 1ß and Toll-like receptor 3 (TLR3) signaling showed impact on APOL1 expression levels, but the predominant effect upon HIV infection is demonstrated for interferons (INF), especially for type 1 INF (INF-α and -β) (32–34). Type 1 INF represents a part of the innate host response against viruses. It induces the intracellular response to viral infection by orchestrating a signaling cascade through the Janus kinase signal transducer and activator of transcription (JAK-STAT) pathway. The JAK-STAT-pathway regulates the transcription of INF-regulated genes (IRG), that contribute to reduce viral spread *via* distinct mechanism like the inhibition of virus entry, translation, replication and viral egress (35).

Additionally, type 1 INF induces apoptosis of infected cells in an autocrine and paracrine manner. Acute and chronic HIV infections constitute a proinflammatory state with elevated levels of interferon in plasma and tissue (36). It was indicated that type 1 INF-α and -β and also type 2 INF γ is able to upregulate APOL1 gene expression in both endothelial cells and podocytes (34). In addition, case reports describe patients carrying APOL1 risk variants who developed cFSGS after treatment with exogenous INF (34, 37) and INF-γ induced proteinuria in APOL1 G1 transgenic mice (38).

Stimulator of interferon genes (STING) is suggested to be an INF-induced candidate pathway that upregulates APOL1. HIV infection induces cyclic guanosine monophosphate-adenosine (cGAMP) synthase (cGAS) to produce cGAMP, an activator of STING (39). Interferon-inducible protein 16 (IFI16) has also been shown to act as a sensor of HIV infection and activator of STING (40). TANK binding kinase 1 (TBK1) is recruited by activated STING and phosphorylates STING and interferon regulatory factor 3 (IRF3). Phosphorylated IRF3 is then translocated to the nucleus and initiates transcription of INF-β and APOL1 in human podocytes. INF-β can furthermore activate the type I IFN receptor, leading to STAT1 phosphorylation through IFNAR-associated JAK1 kinase and increased upregulation of APOL1 and IFI16, further enhancing upregulation of STING (41). Further, cGAS/STING seem to play a key role in the increased endothelial dysfunction mediated by APOL1 risk variants and might contribute to explain the increased sepsis incidence and severity among patients of African ancestry (42).

Both cGAS and IFI16 might also play a role in the progression to lupus nephritis in SLE patients carrying risk alleles of APOL1 (41, 43).

In line with these results, it was demonstrated that retinoic acid-inducible gene I (RIG-I) also recognizes HIV and enhances APOL1 expression and also activation of nuclear factor kappa B (NFкB). The knockdown of RIG-I in human podocytes resulted in attenuated inflammatory and apoptotic effects of APOL1 (44). Additionally, TLR 3 signaling, that can be activated by double stranded RNAs, can be found in replication cycles of nearly all viruses and was shown to enhance APOL1 expression INF-independent *via* NFкB-signaling (34).

To summarize, the described interactions lead to an enhanced expression of APOL1. As the risk variants seem to exert an endotoxic effect on podocytes in a dose-dependent manner, this overexpression can induce podocyte damage in case of HIV infection, whereas, the toxic influence of the risk variants might not be high enough to induce cell damage and therefore cFSGS, without the “second hit” HIV.

Another recently debated mechanism is the contribution of parietal epithelial cells (PEC) to the cFSGS. PECs are capable of self-renewal and differentiation into various cell types, namely, podocytes (45–47) while the regeneration of podocytes appears limited to non-existent (47, 48). It has been shown *in vitro*, that the induction of APOL1 in PECs leads to the expression of podocyte markers, potentially as a repair mechanism and the effort to replace damaged podocytes (49). Furthermore, analysis of the glomerular extracellular matrix shows differences between non-collapsing and cFSGS, with an altered extracellular matrix remodelling and activation of PECs (50).

PEC activation can be detected as a first sign of ensuing glomerular scarring (51). Podocyte hypertrophy, as present in cFSGS, seems to prevent PEC activation and glomerulosclerosis. In glomerular extracts from biopsies of FSGS and diabetic nephropathy mammalian target of rapamycin (mTOR) and PEC-activation related genes were found to be upregulated (48). mTOR-mediated podocyte hypertrophy during podocyte loss seems to be crucial to maintain glomerular functional integrity, as pharmacological mTOR-inhibition during acute podocyte loss resulted in albuminuria, PEC-activation and glomerulosclerosis in mice (48). Interestingly, exacerbated and persistent podocyte hypertrophy also induced podocyte loss and PEC-activation, indicating a limited beneficial effect (48).

These data suggest the targeting of PECs as a potential therapeutic option in cFSGS. APOL1-risk variant effects on podocytes might impair their capacity to prevent PEC-activation and glomerulosclerosis.

## Covid-Associated Nephropathy (COVAN)

Renal involvement with acute kidney injury (AKI), proteinuria and hematuria, worsening the overall prognosis, has been shown frequently in COVID-19 patients (52–54). Chronic kidney disease or conditions with increased risk of impaired kidney function represent strong risk factors for a severe clinical course (55). Biopsy and autopsy studies revealed acute tubular necrosis (ATN) in the majority of COVID-19 associated AKI. Nevertheless, glomerular involvement was also reported and should be distinguished from the majority of AKI due to ATN. Beside reports of minimal change disease, membranous nephropathy, anti-glomerular basement membrane glomerulonephritis, IgA-vasculitis, lupus nephritis and crescentic glomerulonephritis (56–58) associated with SARS-CoV-2, cases of cFSGS displaying similar lesions, observed in HIVAN, have led to the emergence of the SARS-CoV-2 associated entity named “COVAN” (59). COVAN patients mostly present with severe AKI and nephrotic range proteinuria in native kidneys and kidney allografts (60–62).

Like HIVAN, COVAN is mostly reported in patients carrying risk variants of APOL1 supporting the hypothesis of a “second hit” necessary for the onset of APOL1-associated kidney disease. Paying tribute to the short time period of research on COVAN, compared to decades of HIV-research, only few mechanistic data are available for SARS-CoV-2 induced cFSGS. Interestingly, although intracellular and not secreted APOL1 seems to be responsible for kidney injury, a case of SARS-CoV-2 associated cFSGS was reported in a patient carrying 2 APOL1 high risk alleles with a kidney allograft stemming from a low-risk genotype donor (62).

Cytokine induced podocyte damage by the virus and/or direct toxic viral effects on podocytes are suggested to be responsible for SARS-CoV-2 associated cFSGS and might interact with APOL1. The membrane proteins angiotensin converting enzyme II (ACE2) and transmembrane serin protease 2 (TMPRSS2) are used as receptors by SARS-CoV-2 to facilitate cell entry. ACE2 is highly expressed in kidney cells, mainly in the proximal tubule. However, podocytes, parietal epithelial cells, mesangial cells and cells of the collecting duct are found to express ACE2 at lower levels (63). Autopsy studies could detect SARS-CoV-2 RNA and viral proteins in the kidney (64, 65) and indicated, that SARS-CoV-2 renal tropism is associated with the development of acute kidney injury and disease severity (66). In contrast, cases of cFSGS occurred also in patients with mild or even absent respiratory symptoms (67). To our knowledge, all biopsy studies failed to detect SARS-CoV-2 in the kidney. This limits investigating direct toxic viral effects on podocytes and implicates cytokine-mediated effects as shown for HIVAN. Nevertheless, most studies relied on electron microscopy, immunohistochemistry or RNA *in situ* hybridization limiting the conclusions drawn concerning viral detection. PCR based detection methods were regularly able to detect viral RNA within kidney tissue and complimentary RNA and protein detection underline a probable renal SARS-CoV-2 tropism (64, 65, 68–70).

Next to the direct infection of kidney tissue, an injury of the glomerulus induced by the host response might be another plausible mechanism of the development of cFSGS in COVID-19. The often described “cytokine storm” can damage the kidney directly or secondary through the induction of life-threatening circumstances like sepsis, shock, ischemia, hypoxia or rhabdomyolysis (71). Especially patients with severe disease show high plasma levels of cytokines like interleukins, granulocyte cell stimulating factor and tumor necrosis factor α (TNFα) (72). Interferon γ (INF γ) is also upregulated in SARS-CoV-2 infected patients, but a meta-analysis could not show significant differences between the severe and non-severe group of COVID-19 patients (73). Nevertheless, data from subgroups carrying APOL1 risk alleles are not available to our knowledge. A recent study observed that APOL1 risk variants are associated with a higher incidence of sepsis and increased disease severity in patients with COVID-19. Plasma levels of APOL1 were higher in patients with severe sepsis and COVID-19 and correlated with markers of endothelial dysfunction. A mouse model with endothelial cell specific expression of APOL1 risk alleles, developed increased endothelial inflammation, vascular leakage, albuminuria and increased sepsis severity. APOL1 risk variant expression in endothelial cells *in vitro* resulted in mitophagy and leakage of mitochondrial DNA into cytoplasm. Cytosolic DNA is sensed by the NLRP3 inflammasome and by cGAS, an activator of STING, leading to endothelial dysfunction (42). The onset and role of endothelial dysfunction in cFSGS is poorly understood. Although it is commonly assumed that podocyte injury is the first event in the pathogenesis of cFSGS, it was demonstrated, that in Adriamycin-induced nephropathy, endothelial damage occurs prior to podocyte injury (74). Additionally, it was shown that patients with primary FSGS and nephrotic range proteinuria had elevated markers of endothelial dysfunction compared to healthy controls, which were largely related to the activity of the disease (75).

Beyond these recent observations, upregulated cytokines in COVID-19 patients carrying risk alleles of APOL1 could still contribute to the development of cFSGS as a “second hit” as the expression of chemokines (e.g., CCL2 and CXCL10) and Interleukin 6 seems to be elevated within the kidney of COVID 19 patients (76).

It remains elusive, whether podocytes or endothelial cells predominantly orchestrate the histopatholgical changes. All known mechanisms, which lead to kidney damage beyond cFSGS in case of infection with SARS-CoV-2 have been extensively reviewed by Ahmadian et al. (77).

### cFSGS and Other Viral Infections

**Figure 1:**
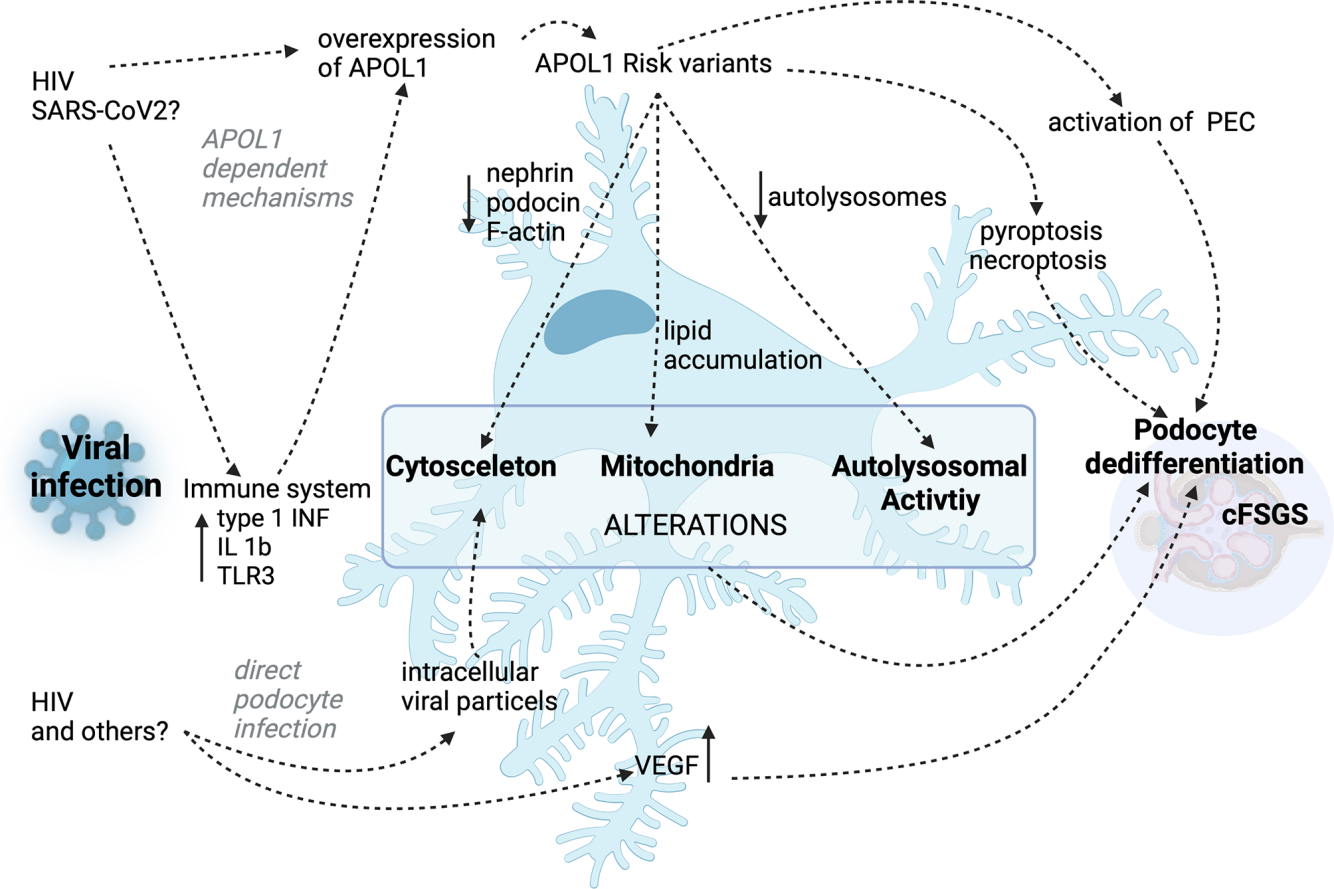
Summary of the proposed APOL1 dependent and APOL1 independent mechanisms in HIV- and other virus-associated cFSGS.

While the association of cFSGS to an infection with HIV is well established and probable for SARS-CoV 2, cases associated with other viral infections are rare and the mechanisms remain incompletely understood due to the limited number of cases. Infections with Parvovirus B19, the cytomegalovirus (CMV), Hepatitis C, simian virus 40 or Epstein–Barr virus are thought to be further potential causes of the development of cFSGS (78). While the CMV seems to be a probable inducer of cFSGS, the association of other viruses and the collapsing variant has still to be proven. Additionally, in 2018 there has been a case series reported the association of dengue virus and zika virus infection and the development of cFSGS (79). In these cases a direct infection of kidney tissue was shown and interestingly, there was no correlation with APOL1 risk alleles. This case study also points to a potential involvement of the complement cascade in the development of the cFSGS. Next to the direct effect of the virus to the podocytes, which has not been shown for these infections, systemic immune response, similar to the response to HIV or SARS-CoV2, might be responsible for upregulation of APOL1 or could directly influence podocyte function (80). A converging mechanism of the innate immune response therefore seems likely, nevertheless has to be further assessed. A summary of these mechanisms is presented in **Figure 1**.

## Conclusion

Research on molecular changes in podocytes expressing risk variants of APOL1 shed light on multiple pathways relevant for podocyte homeostasis. Although none of the suggested pathways alone could explain the development of cFSGS, the role of a second is evident, at least in APOL1 dependent forms. Limited numbers of patients in case reports hamper the identification of “second hits” like HIV, so the pandemic situation of COVID-19 could help to further scientific effort by providing larger cohorts. Furthermore, the analysis of APOL1-risk factor independent cFSGS forms, the identification of viral etiology and associated damaging mechanisms are crucial to understand the development of the disease. Further investigation of the interaction of viral products and the immune response with podocyte signaling pathways that induce the massive morphologic alterations might contribute to our understanding of podocyte biology and the search for targeted therapies in (collapsing) FSGS independent of its etiology.

## Author Contributions

AM and SG have performed intensive studies of the current literature and have written main parts of the text and figure. Both first authors contributed equally to this review. TH and FB have performed extensive correction of the text after studying the literature. TH and FB have contributed significantly with the ideas and structure that are found in the review. All authors contributed to the article and approved the submitted version.

## Funding

TBH was supported by the DFG (CRC1192), and by the BMBF (STOP-FSGS- 01GM1901C).

## Conflict of Interest

The authors declare that the research was conducted in the absence of any commercial or financial relationships that could be construed as a potential conflict of interest.

## Publisher’s Note

All claims expressed in this article are solely those of the authors and do not necessarily represent those of their affiliated organizations, or those of the publisher, the editors and the reviewers. Any product that may be evaluated in this article, or claim that may be made by its manufacturer, is not guaranteed or endorsed by the publisher.
